# Does sustainability marketing make people think food is healthier? A systematic review of health halo effects

**DOI:** 10.1016/j.ssmph.2026.101954

**Published:** 2026-07-27

**Authors:** Agnese Palvarini, David Stuckler, Martin McKee, Courtney McNamara

**Affiliations:** a Imperial College London, Department of Bioengineering, Exhibition Road, London, SW7 2AZ, UK; b Bocconi University, Department of Social and Political Sciences, Via Roentgen, Milan, 20136, Italy; c London School of Hygiene and Tropical Medicine, Department of Health Services Research and Policy, Faculty of Public Health and Policy, Tavistock Place, London, WC1H 9SH, UK; d Newcastle University, Population Health Sciences Institute, Faculty of Medical Sciences, Queen Victoria Road, Newcastle upon Tyne, NE1 4LP, UK

**Keywords:** Sustainability claims, Perceived healthiness, Health halo effects, Commercial determinants of health, Food labelling, Systematic review

## Abstract

**Background:**

Information on sustainability has become increasingly prominent in food environments, shaping how consumers interpret the environmental attributes of food products. This is beginning to attract attention among scholars of the commercial determinants of health as evidence of the effects of sustainability labels and claims on perceptions of healthiness. However, their implications for dietary choices and population health remain fragmented. We report a systematic review examining the relationship between sustainability labels and claims and perceived food healthiness.

**Methods:**

We searched Web of Science and PubMed for empirical studies examining the relationship between sustainability-related product cues and perceived food healthiness. A total of 2714 records were screened, and 233 articles were assessed for eligibility. Of these, 25 studies met our inclusion criteria and were included in the review. We conducted a narrative synthesis of the evidence, analysing patterns across study designs, types of sustainability-related cues, and health-related outcomes.

**Results:**

Across the included studies, sustainability labels and claims frequently increased perceived product healthiness. These effects varied by the type of health-related outcome considered and were more consistently observed in general health perceptions (i.e., food healthiness) than in evaluations of specific nutritional attributes (i.e., calorie content). They also tended to be stronger when these signals were presented in isolation rather than alongside additional product cues.

**Conclusion:**

This review extends the literature on health halo effects to sustainability-related food information and suggests that sustainability labels and claims may influence consumer judgments beyond their intended environmental or ethical purpose. These findings highlight the importance of considering potential spillover effects when designing and regulating sustainability-related information in food environments.

## Highlights

• Sustainability claims create a halo effect by increasing perceived food healthiness.
• Effects are stronger for general health perceptions than for nutritional attributes.
• Informational context shapes the magnitude of these effects.
• Perceptual biases in food environments may act as commercial determinants of health.
• Food labelling regulations should better address sustainability-related marketing.

## 1 Introduction

Understanding why people make the food choices they do and how these choices are often subtly shaped by commercial actors is an urgent challenge for the public health community. Food environments are saturated with signals that influence how consumers perceive and evaluate products, and these cues increasingly shape nutritional judgments in ways that may not reflect underlying nutritional quality (Chandon & Wansink, 2007; Piqueras-Fiszman & Spence, 2015; Swinburn et al., 2011). Experimental evidence consistently shows that perceptions of food healthiness shape dietary behaviours, with products perceived as healthier more likely to be selected or valued more highly, even when not aligned with their nutritional profile (Boncompagni et al., 2025; Chandon & Wansink, 2007; Roe et al., 1999). This micro-level mechanism can scale to population-level effects, as perception-driven dietary behaviours accumulate and shape health outcomes (Afshin et al., 2019; Swinburn et al., 2011).

The commercial determinants of health offer a broader framework for interpreting these dynamics, highlighting how corporate strategies influence the interpretation of food-related information (Gilmore et al., 2023). These pressures are intensifying as food systems undergo rapid transformation, with major implications for both human and planetary health, given the central role of diets in driving global health outcomes and climate change (Willett et al., 2019). Within this context, food marketing and labelling have become powerful forms of exposure, embedding product cues that can steer perceptions of healthiness and influence choice. These perceptual processes are particularly relevant as they can shape food choices, even in the absence of changes in underlying nutritional quality (Gilmore et al., 2023).

Sustainability labels and claims are a prominent example: now widely used to signal environmental or ethical attributes, they represent an evolving and increasingly influential category of product information (Potter et al., 2021). In this review, sustainability labels and claims refer broadly to certified and non-certified sustainability cues displayed on food products, including labels, certifications, scores, logos, and textual claims communicating environmental, climate-related, social, or ethical attributes. These encompass environmental information, including eco-scores, messages linked to recycling or food waste reduction, plant-based or vegan labels, fair-trade certifications, and corporate sustainability practices. While such labels and claims may encourage shifts towards more sustainable diets, they may also shape health perceptions in ways that diverge from nutritional reality, underscoring the need to examine their broader public health impact (Cook et al., 2023).

Prior research shows that consumers often rely on product cues that are not directly related to nutritional composition when forming judgments about food healthiness (Chandon & Wansink, 2007; Schuldt & Schwarz, 2010). These are described as “halo effects” (Thorndike, 1920). When consumers encounter a positive product attribute, such as an environmental or ethical claim, they may generalise this positivity to unrelated domains, including healthiness. This process reflects a form of attribute-based inference, in which a salient cue is used as a shortcut for broader product evaluation (Kahneman, 2011). It can also be understood in terms of dual-process or heuristic reasoning theories, where consumers often rely on fast, intuitive judgments rather than analytic assessment, particularly when evaluating complex attributes such as sustainability or nutrition (Payne et al., 1993; Stanovich & West, 2000). Accordingly, sustainability cues may serve as heuristic signals that simplify evaluation by implying the product's overall “goodness.”

Some sustainability claims may also activate moral halo or moral credentialing effects, whereby ethical or socially responsible attributes lead consumers to view the product as inherently beneficial in other domains (Monin & Miller, 2001). The relationship between sustainability-related cues and perceived healthiness may also reflect a broader conceptual overlap involving naturalness. Previous research suggests that sustainability-related cues can shape consumer inferences beyond their intended environmental meaning, contributing to perceptions of naturalness, quality, and healthiness (Donato et al., 2021; Hallez et al., 2024; Magnier et al., 2016). These associations may be particularly influential because consumers frequently use naturalness as a heuristic for judging food healthiness and safety, reflecting a broader “natural-is-better” bias (Meier et al., 2019; Rozin et al., 2004). This overlap is especially evident in the case of organic labels, which are often interpreted as multidimensional signals encompassing environmental, ethical, and health-related meanings rather than purely sustainability-related attributes (Fernqvist & Ekelund, 2014; Román et al., 2017). Although these labels are relevant within this broader conceptual framework, they were outside the scope of the present review and were therefore excluded, as explained in the Methods section.

For example, environmental sustainability claims may evoke spillover heuristics related to purity or naturalness (Hallez et al., 2024); ethical or fair-trade claims may activate moral credentialing pathways (Schuldt et al., 2012); and circular economy messages may rely more on cognitive associations with waste reduction or resource efficiency (D'Adamo and Colasante, 2022). This tendency is well documented in the context of food labelling and marketing, where similar heuristic processes have been shown to shape health-related judgments. For example, Chandon and Wansink (2007) conducted a series of experimental studies in fast-food settings. They found that health claims led consumers to underestimate calorie content, providing evidence of a “health halo” effect whereby positive product attributes bias health-related expectations.

However, it remains unclear whether similar mechanisms operate in response to sustainability-related product cues. While some studies indicate that ethical or environmental cues displayed on food packaging can increase perceived product healthiness (e.g., Hallez et al., 2024; Paul et al., 2026), others report non-significant or variable effects (e.g., Büttner et al., 2024; Neuhofer et al., 2023). Moreover, existing reviews have examined the broader effects of ecolabels on consumer sustainability perceptions and purchasing behaviours, while largely overlooking their role in shaping perceived food healthiness. For example, Majer et al. (2022) conducted a systematic review of visual sustainability labels across a wide range of psychological and behavioural outcomes, but without a specific focus on food products. Similarly, Andreani et al. (2026) conducted a meta-regression analysis on willingness to pay for sustainability labels and claims in the food industry, without considering health-related perceptions. As a result, existing reviews provide limited insight into whether sustainability-related product cues systematically generate health halo effects, under what conditions these effects emerge, and how they influence health-related judgments. Consequently, the role of sustainability labels and claims in generating health-related inferences remains insufficiently understood, despite growing evidence that these cues may shape consumer evaluations beyond their intended environmental or ethical purpose. To address this gap, we conducted a systematic literature review to examine how sustainability labels and claims influence perceived food healthiness. By synthesising experimental studies that manipulate sustainability-related cues, this review provides a structured assessment of whether, and under what conditions, such signals generate health-related inferences. Unlike existing reviews of sustainability labelling, it focuses exclusively on health-related inferences, distinguishes between general health perceptions and nutrient-specific judgments, and analyses how informational context conditions halo effects. In doing so, it contributes to theory by conceptualising sustainability labels and claims as informational cues that can trigger health-related inferences within food environments, identifying the conditions under which these effects are more likely to emerge, and situating these processes within the broader framework of Commercial Determinants of Health.

## 2 Methods

### 2.1 Review design and protocol

We conducted a systematic literature review to examine how sustainability labels and claims shape consumers’ perceptions of food healthiness, following the Preferred Reporting Items for Systematic Reviews and Meta-Analyses (PRISMA) guidelines (Page et al., 2021). Although originally developed within medical and public health research, PRISMA has increasingly been adopted in food, sustainability, and consumer behaviour research to ensure transparency and replicability in evidence synthesis (Andreani et al., 2026; Branca et al., 2024; Potter et al., 2021; Steiner & Florack, 2023). Our protocol was registered in PROSPERO (CRD420261328242).

### 2.2 Search strategy

We searched Web of Science Core Collection (Topic Field) and PubMed (Title/Abstract fields) on 16 February 2026 for empirical studies examining the relationship between sustainability labels and claims and perceived food healthiness. Previous systematic reviews in related fields have adopted different database combinations depending on their specific objectives and disciplinary focus. For example, reviews examining willingness to pay for sustainability labels (Andreani et al., 2026), sustainable packaging and consumer perspectives (Branca et al., 2024), the effects of environmental sustainability labels on food-related behaviours (Potter et al., 2021), and organic halo effects on food evaluations (Durand et al., 2025) employed different search strategies and database selections. Consistent with the interdisciplinary nature of the present review, Web of Science and PubMed were selected because together they capture the core disciplines relevant to the study. Web of Science provides broad coverage of behavioural, marketing, and environmental research, while PubMed indexes the leading literature on nutrition, health perception, and public health. Given that sustainability-related health inferences sit at this intersection, these two databases offer comprehensive and efficient coverage of the most pertinent empirical studies. Grey literature was excluded to ensure that all included studies met consistent standards of methodological transparency and peer-reviewed quality. This approach is consistent with previous systematic reviews in related fields that similarly restricted their evidence base to peer-reviewed research (Andreani et al., 2026; Branca et al., 2024; Steiner & Florack, 2023). Because the analysis focuses on experimentally derived evidence of perceptual effects, limiting the search to peer-reviewed sources reduces variability in study design, reporting, and measurement. This approach also enhances reproducibility by relying on literature indexed in established academic databases rather than unpublished or non-standardised material.

The search strategy was structured around three core keyword clusters: health-related perceptions, sustainability labels and claims, and food products. These clusters were developed by adapting terminology from previously published systematic reviews examining food healthiness perceptions (Plasek et al., 2020; Steiner & Florack, 2023), sustainability labels and claims (Andreani et al., 2026; Branca et al., 2024; Potter et al., 2021), and food-related evaluations and halo effects (Andreani et al., 2026; Durand et al., 2025). Methodological terms related to study design were not included in the search strategy. This decision was made to maximise search sensitivity and minimise the risk of excluding relevant studies, as experimental and quantitative research is not always indexed using consistent methodological terminology. Instead, eligibility criteria related to study design were applied during the screening and full-text assessment stages. The search remained focused because it required the simultaneous presence of terms related to health perceptions, sustainability, and food products, substantially reducing the number of potentially relevant records retrieved.

Specific search terms within each cluster were adapted from previously published systematic reviews. For health-related keywords, we referred to Andreani et al. (2026), Plasek et al. (2020), and Steiner and Florack (2023). For sustainability-related keywords, we referred to Andreani et al. (2026). For food-related keywords, we referred to Andreani et al. (2026) and Durand et al. (2025). Health- and nutrition-labelling terminology was included within the health-related keyword cluster to capture studies examining how sustainability-related cues influence perceived food healthiness and nutritional evaluations. Preliminary scoping of the literature indicated that such terminology was frequently used in this body of research. This resulted in the following search string:

a) “Health* label*” OR “Nutri* label*” OR “Health* claim*” OR “Nutri* claim*” OR Healthiness OR Healthfulness OR “Health halo*” OR “Halo effect*” AND
b) Sustainab* OR Environment* OR “Fair trade” OR Eco* OR Bio* OR Organic* OR Green* OR Recycl* AND
c) Food OR Meat OR Dairy OR Fruit* OR Vegetable* OR Bread OR Pasta OR Wine OR Oil* OR Seed* OR Grain* OR Egg* OR Juice* OR Milk OR Cheese* OR Yogurt OR Ham OR Calories

The verbatim search string for each database is reported in Supplementary Material 1.

**Fig. 1:**
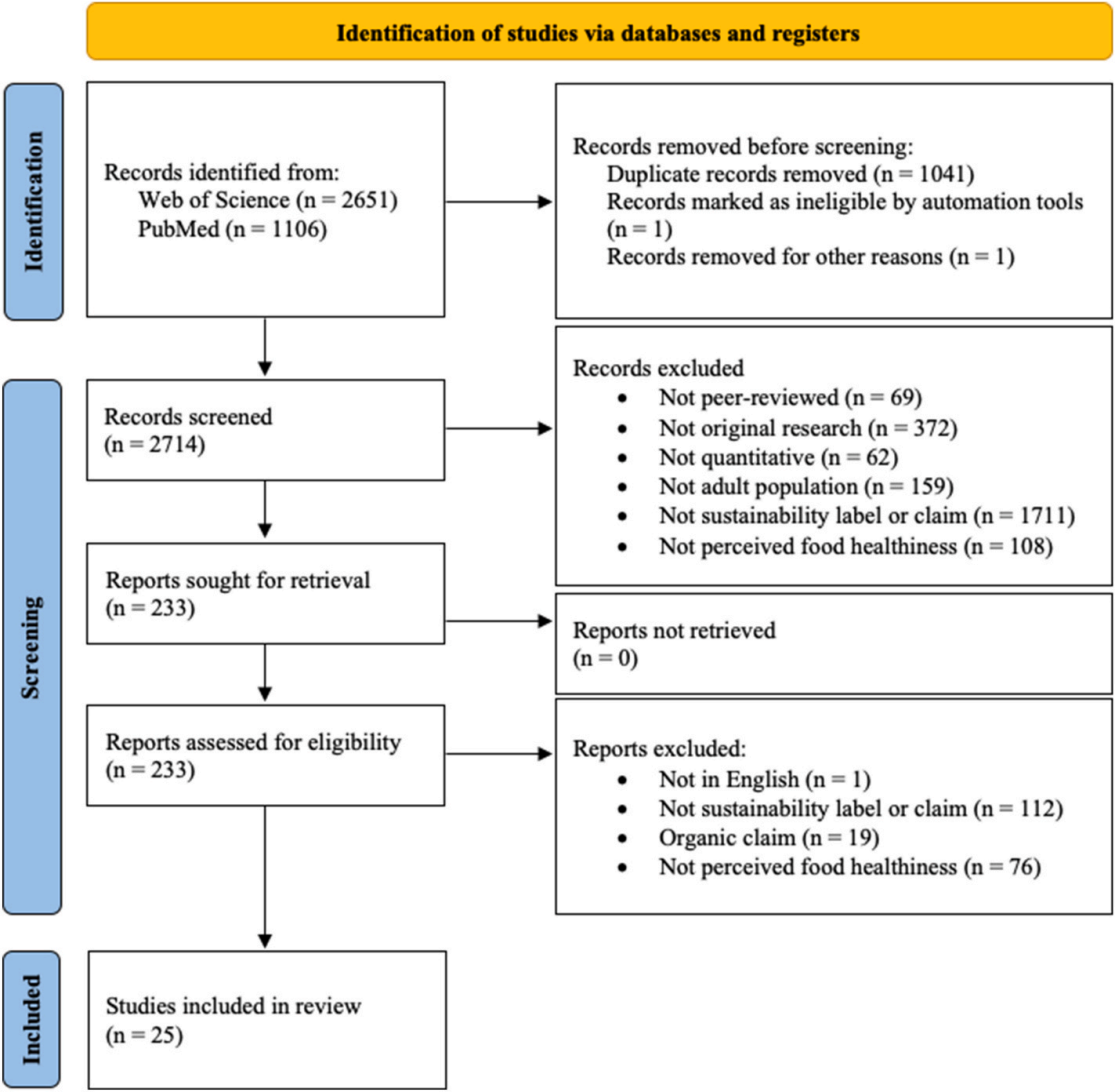
PRISMA flow diagram of the study selection process.

The search yielded 2651 records from Web of Science and 1106 from PubMed, for a total of 3757 records. All records were imported into Zotero, which identified 1041 articles as duplicates. After removing these, Zotero marked one record as ineligible because it corresponded to a paper that had been withdrawn from publication. Another record was removed because it was the retraction notice for the above-mentioned paper. At the end, we were left with 2714 articles for the screening and eligibility stages. Fig. 1 depicts the PRISMA flow inclusion diagram of the study selection process, while the PRISMA checklist is provided in Supplementary Material 2.

### 2.3 Inclusion and exclusion criteria

Eligibility criteria were defined a priori using the PICO framework. Studies were included if they:

a) were original contributions published in peer-reviewed journals;
b) employed experimental, quasi-experimental, or quantitative survey-based designs;
c) involved adult consumers (≥18 years); and
d) tested the relationship between sustainability labels and claims and health-related food perceptions.

Studies were excluded if they were not peer-reviewed; consisted of non-original publications (e.g., reviews, editorials, book chapters); employed qualitative or descriptive designs; focused exclusively on children, adolescents, or clinical populations; examined general sustainability attitudes or non-food environmental labels; or assessed only purchase intention, willingness to pay, or sustainability perceptions. The review was restricted to adult consumers to improve comparability across studies and provide a more homogeneous context for examining health-related inferences arising from sustainability labels and claims. Although children and adolescents represent important and vulnerable populations whose food-related perceptions and behaviours can also be influenced by marketing practices and packaging cues (Enax et al., 2015; Harris et al., 2021; Lapierre et al., 2017), they differ from adults in several aspects relevant to food decision-making, including cognitive development, nutrition literacy, autonomy over food choices, and the extent to which decisions are mediated by their parents or caregivers (Ares et al., 2024; Bassett et al., 2008; Mura Paroche et al., 2017; Scaglioni et al., 2018; Zarnowiecki et al., 2012). These developmental and contextual differences are likely to influence how food-related information is processed and interpreted, thereby introducing substantial conceptual and methodological heterogeneity (Albardan & Platat, 2025; Serrano-Gonzalez et al., 2021). The review therefore focused exclusively on adults to maintain greater comparability across studies while recognising that the effects of sustainability labels and claims in younger populations warrant dedicated investigation. No restrictions were applied based on publication year or geographic location. Language restrictions were not applied at the search stage; however, non-English publications were excluded during the full-text eligibility assessment.

### 2.4 Screening and eligibility workflow

Screening and eligibility assessment were initially performed by AP. To ensure consistency, a random sample of 10% of records was independently reviewed by CM and DS. In addition, all ambiguous records and uncertain eligibility decisions were discussed among the three authors involved in the review process. Any discrepancies were resolved through discussion until consensus was reached. Of the 2714 records screened, 2481 were excluded for the following reasons: not examining sustainability labels or claims (n = 1711), not original research (n = 372), not involving adult populations (n = 159), not assessing perceived food healthiness (n = 108), not peer-reviewed (n = 69), or not quantitative design (n = 62). A total of 233 reports were retrieved for full-text assessment.

Full-text eligibility screening resulted in the exclusion of 208 studies. Of these, 112 did not manipulate or examine sustainability labels or claims, 76 did not assess health-related inferences, and one was not in English. In addition, 19 studies focusing exclusively on organic claims were excluded. Organic labelling occupies a hybrid conceptual position at the intersection of sustainability and health (Fernqvist & Ekelund, 2014; Hughner et al., 2007). While organic claims can be linked to environmentally oriented production practices, consumers frequently associate them with a broader set of meanings related to naturalness, purity, food safety, and product quality (Durand et al., 2025; Fernqvist & Ekelund, 2014; Hughner et al., 2007; Magnusson et al., 2001; Román et al., 2017). Unlike purely sustainability-related cues, organic labels and claims are intrinsically associated with personal health benefits in consumer interpretations (Shaikh et al., 2024). Consequently, health-related inferences attributable to the sustainability dimension of organic labels and claims are not readily distinguishable from those arising from pre-existing beliefs attached to organic products (Fernqvist & Ekelund, 2014; Schuldt & Schwarz, 2010; Shaikh et al., 2024). Furthermore, organic labelling is governed by a dedicated regulatory framework that distinguishes it from broader sustainability labels and claims (FAO and WHO, 2007). Given this conceptual overlap, together with the specific certification schemes governing organic food and the breadth and volume of dedicated scholarship, the organic labelling literature warrants a separate systematic review. A total of 25 studies met all inclusion criteria and were included in the final analytical sample. The retained studies exclusively manipulated sustainability-related cues to evaluate their influence on perceived food healthiness. The study selection process is summarised in Fig. 1.

### 2.5 Data extraction and synthesis

We extracted information on author, year, country, population, sample size, study design, and experimental setting. We also collected data on the intervention, including the type of sustainability-related cue, the presence of additional claims, and the control condition, as well as on outcomes related to perceived food healthiness, the measurement tools used, and the reported effects. To ensure consistency, selected variables were classified using predefined criteria. A summary of the analytical categories used in the review is provided in Table 1.

**Table 1:** Analytical categories used for outcome and effect classification.

Analytical domain	Category	Sub-category	Description
Health outcome category	Health perceptions	–	General evaluations of a product's health implications, including perceived healthiness, healthfulness, safety, naturalness, quality, health properties, or health risk.
Health outcome category	Nutrition perceptions	–	Perceptions of specific nutritional attributes, including calorie, fat, sodium, protein, vitamin content, nutritional value, or nutritiousness.
Effect typology	Robust effects	Positive effect	Sustainability-related cue increases perceived healthiness.
		Negative effect	Sustainability-related cue decreases perceived healthiness.
		Opposite effect	Outcome direction follows sustainability-related cue valence (i.e., a favourable sustainability-related cue increases perceived healthiness, whereas an unfavourable one decreases it).
Effect typology	Null effects	Non-significant effect	Observed differences likely due to chance rather than the experimental manipulation.
		Negligible effect	Differences numerically very small and not indicative of a meaningful relationship.
Effect typology	Heterogeneous effects	Partial effect	Only some comparisons within the same outcome are significant.
		Inconsistent effect	Contradictory or non-interpretable findings.
		Mixed effect	Different outcomes within the same study show different effect directions.

Outcome measures of perceived food healthiness were classified into analytical categories. As several studies reported multiple outcomes, the analysis was conducted at the outcome-level rather than the study-level, resulting in 43 observations across 25 studies. Outcomes were grouped into two categories. Health perception includes general evaluations of a product's health implications, such as perceived healthiness, healthfulness, or health-related judgments. Nutrition perception refers to perceptions of specific nutritional attributes, perceived nutritional value, or nutritiousness.

Effects were grouped into three macro-categories: robust, null, and heterogeneous. Robust effects include positive, negative, and opposite patterns. Null effects include non-statistically significant and negligible results. Heterogeneous effects include partial, inconsistent, and mixed patterns.

The methodological quality of the included studies was assessed using the Joanna Briggs Institute (JBI) critical appraisal tools, selecting the appropriate checklist based on study design. Responses were recorded as “yes”, “no”, “unclear”, or “not applicable”, and studies were classified as low, moderate or high risk of bias. Studies were classified as low risk when they were randomised controlled trials with clearly reported randomisation procedures and representative samples. Studies were classified as moderate risk when they involved experimental manipulation of claims but showed less transparent randomisation procedures. Studies were classified as high risk when outcome measurement was less reliable or potentially biased. Uncertain cases were cross-checked among three researchers. No studies were excluded based on quality assessment. Full results of the risk of bias assessment are reported in Supplementary Materials 3, 4, and 5.

The findings were integrated in narrative form due to the substantial heterogeneity among the included studies. Sustainability labels and claims varied widely in type (e.g., environmental, ethical, circular economy messages), and outcomes ranged from global healthiness judgments to specific nutrient estimates, each measured using different scales and metrics. Studies also differed in presentation contexts. Some tested claims in isolation, while others tested them alongside nutrition labels or warnings, making effect sizes non-comparable. Because these dimensions intersect in inconsistent ways, there was no coherent set of studies with sufficiently aligned interventions, outcomes, and measurement units to support reliable pooling.

## 3 Results

### 3.1 Characteristics of the included studies (n = 25)

Of the 25 included experimental studies, most were published in recent years, with more than half published from 2024 onwards (n = 13). This recent growth likely reflects the increasing prominence of sustainability communication in food systems and the growing interest in understanding its potential implications for consumer health-related judgments. Geographically, the evidence base was concentrated in Europe (n = 13) and North America (n = 8), with most conducted within a single-country context (n = 24). This geographical concentration may reflect the stronger institutional development of sustainability labelling schemes and consumer research infrastructures in these regions, while also highlighting the limited evidence currently available from low- and middle-income countries.

Most studies examined adult consumer samples (n = 19), with fewer focusing on university students (n = 3) or representative population samples (n = 3). The predominance of online experimental designs may reflect their efficiency in testing consumer responses to information cues under controlled conditions, although this may come at the expense of ecological validity compared with real-world purchasing environments. Randomised designs predominated (n = 19), whereas six studies relied on non-randomised approaches. Most were conducted in online environments (n = 20), with a few in laboratory (n = 3) or field (n = 2) settings.

Sustainability labels and claims spanned a wide range of formulations and, in most cases, were presented without additional cue manipulations (n = 15). In terms of outcomes, most studies examined health perception outcomes (n = 18), while a smaller number assessed both health and nutrition perceptions (n = 6), and only one study focused exclusively on nutrition perception outcomes. Perceived healthiness was typically measured using rating scales (n = 23), such as Likert-type, Semantic Differential, and Visual Analogue Scales.

The majority of studies were rated as having a moderate risk of bias (n = 22), with only two classified as low risk and one as high risk.

Table 2 summarises the main characteristics of the 25 studies included in the review.

**Table 2:** Characteristics of the studies included in the review.

Study	Country	Population	Sample size	Study design	Sustainability label or claim	Additional cues	Outcome	Measurement tool	Risk of bias
Berry and Romero (2021)	USA	Adult consumers	142	Randomised online experiment	Fair trade label	No	Health perception	7-point rating scale	Moderate
Biondi and Camanzi (2020)	Italy	Adult consumers	1231	Randomised online experiment	Circular economy claim	Yes	Health perception	Rating scale (from −5 to +5)	Moderate
Bschaden et al. (2022)	Germany	Adult consumers	214	Non-randomised field experiment	Food waste information	No	Health and nutrition perception	Multiple measurement tools	Moderate
Büttner et al. (2024)	Germany	Representative sample	1010	Randomised online experiment	Eco-Score	No	Health and nutrition perception	7-point rating scale	Moderate
Carneiro et al. (2025)	Germany	Adult consumers	203	Randomised online experiment	Rescued claim	No	Health perception	7-point rating scale	Moderate
Caso et al. (2023)	Italy	Adult consumers	150	Non-randomised laboratory experiment	Sustainable agriculture certification	No	Health and nutrition perception	7-point rating scale	Moderate
Dervishi and Dohle (2025)	Germany	Adult consumers	1080	Randomised online experiment	Eco-Score	Yes	Health perception	Rating scale (from 0 to 100)	Moderate
Eichin et al. (2025)	Austria	Adult consumers	712	Randomised online experiment	Sustainability score	Yes	Health perception	10-point rating scale	Moderate
Grummon et al. (2025)	USA	Adult consumers	3147	Randomised online experiment	Environmentally friendly label	No	Health perception	5-point rating scale	Low
Hallez et al. (2024)	Belgium	Adult consumers	221	Non-randomised online experiment	Recycled content claim	No	Health perception	6-point rating scale	Moderate
Jürkenbeck et al. (2024)	Germany	Adult consumers	1061	Randomised online experiment	Eco-Score	Yes	Health perception	Rating scale (from 0 to 100)	Moderate
Kimura et al. (2010)	Japan	University students	151	Randomised laboratory experiment	Carbon footprint score	No	Health perception	5-point rating scale	Moderate
Lieke et al. (2024)	Germany	Adult consumers	411	Non-randomised online experiment	Sustainability certification	Yes	Health perception	% of respondents	High
Marquis et al. (2023)	Colombia and France	University students	296	Non-randomised online experiment	Environmental claim	Yes	Health perception	9-point rating scale	Moderate
Mediano et al. (2026)	Chile	Adult consumers	611	Randomised online experiment	Chilean eco-label; Fictitious environmental warning label	Yes	Health and nutrition perception	5-point rating scale	Moderate
Neuhofer et al. (2023)	USA	Representative sample	2000	Randomised online experiment	Sustainability Facts Label (SFL)	Yes	Health and nutrition perception	5-point rating scale	Moderate
Paul et al. (2026)	USA	University students	144	Randomised laboratory experiment	Green energy label	No	Health perception	7-point rating scale	Moderate
Pink et al. (2022)	USA	Adult consumers	901	Randomised online experiment	Carbon footprint score	Yes	Health perception	Rating scale (from 0 to 100)	Moderate
Ruan et al. (2026)	China	Adult consumers	200	Randomised online experiment	Eco-friendly packaging	No	Health perception	7-point rating scale	Moderate
Schuldt et al. (2012)	USA	Adult consumers	56	Randomised online experiment	Fair trade claim	No	Nutrition perception	7-point rating scale	Moderate
Shaikh et al. (2024)	France	Adult consumers	577	Randomised online experiment	Eco-Score	Yes	Health perception	7-point rating scale	Moderate
Stremmel et al. (2022)	Germany	Adult consumers	398	Randomised online experiment	Vegan label	No	Health perception	7-point rating scale	Moderate
Swahn et al. (2025)	Sweden	Adult consumers	329	Non-randomised field experiment	Vegan label	No	Health perception	Rating scale (from 0 to 100)	Moderate
Wei et al. (2018)	USA	Adult consumers	520	Randomised online experiment	Corporate Social Responsibility (CSR) claims	No	Health and nutrition perception	7-point rating scale	Moderate
Wolfson et al. (2022)	USA	Representative sample	5049	Randomised online experiment	Climate impact label	No	Health perception	7-point rating scale	Low

### 3.2 Association of sustainability labels and claims with perceived healthiness (n = 43)

Following the classification framework described in the Methods section, outcomes were grouped into three macro-categories: robust, null, and heterogeneous effects, with results showing a predominance of positive or null patterns.

Robust effects were the most frequent (n = 24; 55.81% of outcomes), followed by null effects (n = 14; 32.56%) and a smaller number of heterogeneous results (n = 5; 11.63%). Within the robust category, effects were predominantly positive (n = 19), indicating that sustainability-related information often increased the perceived healthiness of food products. Four outcomes reflected opposite patterns, while one outcome showed a negative association. Null effects mainly reflected non-statistically significant results (n = 12) and two negligible effects. The remaining outcomes showed heterogeneous patterns, mostly associated with partial effects (n = 4) or inconsistent effects (n = 1).

**Fig. 2:**
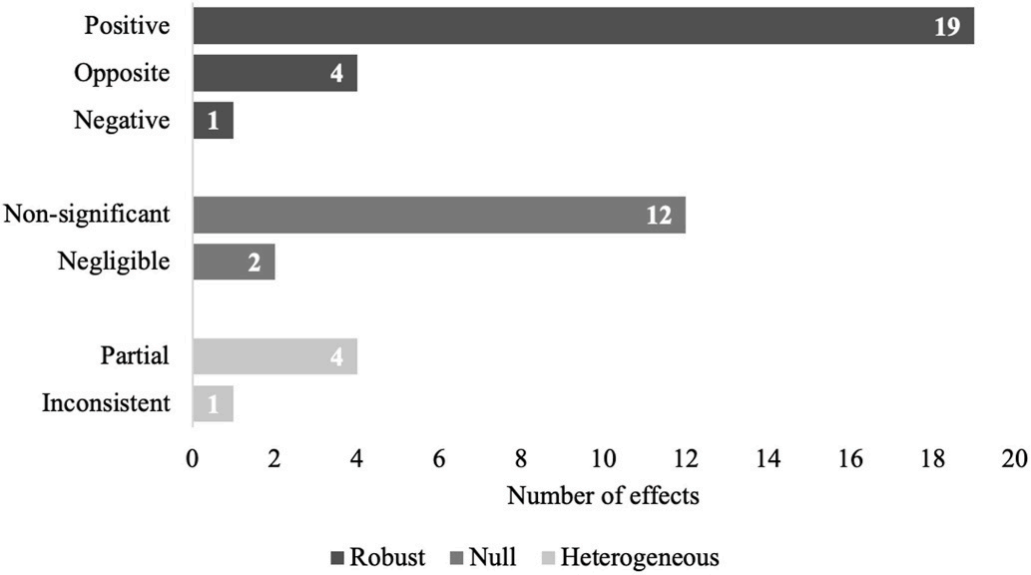
Distribution of outcome-level effects.

Fig. 2 presents the distribution of these effects across the different effect categories.

### 3.3 Association of sustainability labels and claims with health-related perceptions (n = 30)

When focusing on health-perception outcomes, a clear and consistent pattern emerges: sustainability labels and claims frequently increase the perceived healthiness of food products. Of the 30 outcomes assessing perceived healthiness or related health judgments, positive effects were most frequent (n = 15; 50.00% of outcomes). Non-statistically significant effects were the second most common pattern (n = 8; 26.67%), while other effect types were comparatively rare, including opposite and negligible effects in two studies each, and negative, partial, and inconsistent results in one study each. This pattern is also reflected in the broader classification of effects, where robust effects predominated (n = 18; 60.00%), compared with null effects (n = 10; 33.33%) and heterogeneous effects (n = 2; 6.67%).

Several studies consistently reported that sustainability-related cues increased perceived healthiness across different product categories, sustainability attributes, and experimental settings. Positive effects were observed across a range of sustainability labels and claims, suggesting that consumers frequently generalise sustainability-related information into broader evaluations of product healthiness. However, this tendency was not universal, as some studies showed that the direction of the effect depended on the valence and framing of the sustainability-related cue. Evidence of the overall positive pattern was provided by Hallez et al. (2024), who conducted an online experiment with young adults in Belgium (n = 221) manipulating packaging material (glass vs. plastic) and a recycled content claim (”100% recycled”). Health-related perceptions—healthiness, naturalness, and quality—were all higher in the presence of the claim, suggesting a positive halo effect of recycled packaging. Similar findings were reported in a randomised laboratory experiment in the United States by Paul et al. (2026) involving undergraduate students (n = 144). Participants evaluated a bag of potato chips with or without a green energy label (“Made with Renewable Wind and Solar Energy”) and rated its perceived healthiness. The sustainability claim increased perceived healthiness, with labelled products rated as healthier.

At the same time, some studies highlighted important boundary conditions. This was observed by Mediano et al. (2026), who conducted an online experiment in Chile where participants evaluated food packages displaying a calorie warning alongside different environmental labels (none, a positive ecolabel, or an environmental warning). Environmental labels significantly influenced perceived healthiness: products with an environmental warning were rated as less healthy than those with no label or a positive ecolabel. In contrast, environmental labels did not significantly affect perceived health risk.

The distribution of effect categories across health-related perception outcomes is illustrated in Table 3.

**Table 3:** Distribution of effects across health-related perception outcomes.

Study	Outcome	Effect	Results
Berry and Romero (2021)	Perceived healthiness	Positive	Fair trade label increases perceived healthiness (M_EG_ = 4.55 vs. M_CG_ = 3.93; p = 0.025).
Biondi and Camanzi (2020)	Perceived healthiness	Non-statistically significant	Post-hoc comparisons indicate no statistically significant effect of “Full resource exploitation in a circular economy perspective” claim on perceived healthiness.
	Perceived naturalness	Non-statistically significant	Post-hoc comparisons indicate no statistically significant effect of “Full resource exploitation in a circular economy perspective” claim on perceived naturalness.
Bschaden et al. (2022)	Perceived healthiness	Non-statistically significant	Non-statistically significant effect of “One third of food produced worldwide is wasted […]” information on perceived healthiness (p = 0.902).
Büttner et al. (2024)	Perceived healthiness	Opposite	Positive Eco-Score increases perceived healthiness (e.g., spaghetti: M_EG_ = 4.81 vs. M_CG_ = 4.44; p < 0.01), whereas negative Eco-Score decreases it (M_EG_ = 3.87 vs. M_CG_ = 4.44; p < 0.01).
Carneiro et al. (2025)	Perceived healthiness	Positive	“Rescued foods are made from ingredients that would otherwise go to waste […]” claim increases perceived healthiness (M_EG_ = 3.95 vs. M_CG_ = 3.26; p < 0.001).
Caso et al. (2023)	Perceived safety	Positive	“With flour from sustainable agriculture” certification increases perceived safety (M_EG_ = 5.85 vs. M_CG_ = 5.63; p = 0.030).
	Perceived health properties	Positive	“With flour from sustainable agriculture” certification increases perceived health properties (M_EG_ = 5.85 vs. M_CG_ = 5.57; p < 0.001).
Dervishi and Dohle (2025)	Perceived healthiness	Positive	Positive Eco-Score increases perceived healthiness (b = 2.54; p = 0.001).
Eichin et al. (2025)	Perceived healthiness	Positive	High sustainability score increases perceived healthiness (M_H_ = 6.44 vs. M_L_ = 6.06; β = 0.22; CI [0.20–0.24]).
Grummon et al. (2025)	Perceived healthiness	Positive	Environmentally friendly label increases perceived healthiness (M_EG_ = 2.96 vs. M_CG_ = 2.84; p = 0.005).
Hallez et al. (2024)	Perceived healthiness	Positive	“100% recycled” claim increases perceived healthiness (b = 0.11; p < 0.01).
	Perceived naturalness	Positive	“100% recycled” claim increases perceived naturalness (b = 0.11; p < 0.05).
	Perceived quality	Positive	“100% recycled” claim increases perceived quality (b = 0.48; p < 0.001).
Jürkenbeck et al. (2024)	Perceived healthiness	Partial	Positive Eco-Score increases perceived healthiness (e.g., fries: M_EG_ = 39.86 vs. M_CG_ = 27.63; p < 0.001), whereas negative Eco-Score shows no statistically significant effect (p > 0.05).
Kimura et al. (2010)	Perceived healthiness	Non-statistically significant	Non-statistically significant effect of carbon footprint score on perceived healthiness (p > 0.05).
	Perceived quality	Non-statistically significant	Non-statistically significant effect of carbon footprint score on perceived quality (p > 0.05).
Lieke et al. (2024)	Perceived healthiness	Negative	Certified Sustainable Palm Oil label is associated with a decrease in perceived healthiness (e.g., chocolate: N_SC_ = 11.9% vs. N_HC_ = 34.1%).
Marquis et al. (2023)	Perceived healthiness	Negligible	“Saving the planet” claim shows no change in perceived healthiness (e.g., chips: Med_EG_ = 5 vs. Med_CG_ = 5).
Mediano et al. (2026)	Perceived healthiness	Opposite	“More than 80% recyclable” label increases perceived healthiness (M_EG_ = 2.8 vs. M_CG_ = 2.7; p = 0.001), whereas “High environmental impact” label decreases it (M_EG_ = 2.3 vs. M_CG_ = 2.7; p = 0.001).
	Perceived health risk	Non-statistically significant	Non-statistically significant effect of “More than 80% recyclable” and “High environmental impact” labels on perceived health risk (p = 0.061).
Neuhofer et al. (2023)	Perceived safety	Non-statistically significant	Non-statistically significant effect of Sustainability Facts Label on perceived safety (p > 0.05).
Paul et al. (2026)	Perceived healthiness	Positive	“Made with Renewable Wind and Solar Energy” label increases perceived healthiness (M_EG_ = 2.71 vs. M_CG_ = 2.14; p = 0.008).
Pink et al. (2022)	Perceived healthiness	Inconsistent	Carbon footprint score decreases perceived healthiness in some cases (e.g., moderately harmful foods: M_EG_ = 58.89 vs. M_CG_ = 60.58; p < 0.001) but increases it in others (e.g., harmful foods: M_EG_ = 38.67 vs. M_CG_ = 36.94; p < 0.001).
Ruan et al. (2026)	Perceived healthiness	Positive	Eco-friendly packaging increases perceived healthiness (M_EG_ = 5.53 vs. M_CG_ = 3.99; p < 0.001).
Shaikh et al. (2024)	Perceived healthiness	Non-statistically significant	Non-statistically significant effect of Eco-Score on perceived healthiness (p = 0.313).
Stremmel et al. (2022)	Perceived healthiness	Positive	Vegan label increases perceived healthiness (e.g., spread with herbs: M_EG_ = 4.8 vs. M_CG_ = 3.9; p = 0.022).
Swahn et al. (2025)	Perceived healthiness	Positive	Vegan label increases perceived healthiness compared with plant-based label (β = 3.28; p = 0.05).
Wei et al. (2018)	Perceived healthiness	Positive	Corporate Social Responsibility claims increase perceived healthiness (e.g., food manufacturing claim: M_EG_ = 3.947 vs. M_CG_ = 3.241; p < 0.001).
Wolfson et al. (2022)	Perceived healthiness	Negligible	Climate impact labels show no consistent effect on perceived healthiness across food products.

### 3.4 Association of sustainability labels and claims with nutrition-related perceptions (n = 13)

A more heterogeneous and less consistent pattern emerges when examining nutrition perception outcomes. Among the 13 outcomes assessing specific nutritional attributes, results were more evenly distributed across effect categories. Positive effects (n = 4) and non-statistically significant effects (n = 4) occurred with equal frequency, while partial effects were observed in three studies and opposite effects in two. Consistent with this distribution, robust effects were less dominant (n = 6; 46.15% of outcomes), compared with null (n = 4; 30.77%) and heterogeneous effects (n = 3; 23.08%).

The evidence suggests that sustainability labels and claims influence nutrition-related perceptions less consistently than general health perceptions. While some studies found no effect on nutritional evaluations, others reported effects limited to specific nutrients, indicating that sustainability-related information does not systematically translate into nutritional inferences. This variability is reflected in the findings of Caso et al. (2023), who conducted a laboratory experiment in Italy with adult consumers responsible for household food shopping (n = 150). Participants evaluated two identical cookie packages, one labelled “sustainable agriculture” and one unlabelled. No statistically significant differences emerged in perceived nutritional properties, indicating that the sustainability claim did not affect perceptions of nutritional characteristics. Other studies reported more mixed patterns across nutritional attributes, such as Neuhofer et al. (2023), who conducted an online experiment with a nationally representative sample of U.S. milk consumers (n = 2000). Participants evaluated organic and conventional milk under different labelling conditions and reported beliefs about product attributes. Sustainability-related information did not significantly affect perceived calorie content, but it had a small positive effect on protein expectations, with organic milk perceived as having slightly more protein.

At the same time, a smaller group of studies reported more pronounced health halo effects, indicating that sustainability-related cues can influence specific nutritional judgments under certain conditions. Stronger effects were reported by Schuldt et al. (2012), who conducted an online experiment with U.S. adults (n = 56) in which participants evaluated a chocolate product labelled “fair trade” or unlabelled. Chocolate labelled as fair trade was perceived as having fewer calories, indicating that a social-ethics label can generate a positive spillover on calorie expectations.

The distribution of effect categories across nutrition-related perception outcomes is illustrated in Table 4.

**Table 4:** Distribution of effects across nutrition-related perception outcomes.

Study	Outcome	Effect	Results
Bschaden et al. (2022)	Perceived calorie content	Non-statistically significant	Non-statistically significant effect of “One third of food produced worldwide is wasted […]” information on perceived calorie content (p = 0.277).
Büttner et al. (2024)	Perceived nutritiousness	Partial	Negative Eco-Score decreases perceived nutritiousness (e.g., spaghetti: M_EG_ = 4.32 vs. M_CG_ = 4.68; p < 0.05), whereas positive Eco-Score shows no statistically significant effect (e.g., spaghetti: p > 0.05).
	Perceived fat content	Partial	Negative Eco-Score increases perceived fat content (e.g., yogurt: M_EG_ = 3.77 vs. M_CG_ = 4.09; p < 0.05), whereas positive Eco-Score shows no statistically significant effect (e.g., yogurt: p > 0.05).
	Perceived calorie content	Partial	Negative Eco-Score increases perceived calorie content (e.g., yogurt: M_EG_ = 3.70 vs. M_CG_ = 4.00; p < 0.05), whereas the effect is not statistically significant across all food products (e.g., spaghetti: p > 0.05).
	Perceived vitamin content	Opposite	Positive Eco-Score increases perceived vitamin content (e.g., yogurt: M_EG_ = 4.19 vs. M_CG_ = 3.79; p < 0.01), whereas negative Eco-Score decreases it (e.g., yogurt: M_EG_ = 3.41 vs. M_CG_ = 3.92; p < 0.01).
Caso et al. (2023)	Perceived nutritional properties	Non-statistically significant	Non-statistically significant effect of “With flour from sustainable agriculture” certification on perceived nutritional properties (p = 0.288).
Mediano et al. (2026)	Perceived calorie content	Non-statistically significant	Non-statistically significant effect of “More than 80% recyclable” and “High environmental impact” labels on perceived calorie content (p = 0.159).
	Perceived sodium content	Opposite	“More than 80% recyclable” label decreases perceived sodium content (M_EG_ = 3.0 vs. M_CG_ = 3.2; p = 0.006), whereas “High environmental impact” label increases it (M_EG_ = 3.3 vs. M_CG_ = 3.2; p = 0.006).
Neuhofer et al. (2023)	Perceived calorie content	Non-statistically significant	Non-statistically significant effect of Sustainability Facts Label on perceived calorie content (p > 0.05).
	Perceived protein content	Positive	Sustainability Facts Label increases perceived protein content (Δ = 0.071; p < 0.05).
Schuldt et al. (2012)	Perceived calorie content	Positive	“Petersen's pays its cocoa farmers 50% more than the standard market price for cocoa […]” claim decreases perceived calorie content (M_EG_ = 4.30 vs. M_CG_ = 4.76; p = 0.03).
Wei et al. (2018)	Perceived nutritional value	Positive	Corporate Social Responsibility claims increase perceived nutritional value (e.g., food manufacturing claim: M_EG_ = 3.797 vs. M_CG_ = 3.323; p < 0.05).
	Perceived calorie content	Positive	Corporate Social Responsibility claims decrease perceived calorie content (e.g., food manufacturing claim: M_EG_ = 3.791 vs. M_CG_ = 4.288; p < 0.001).

### 3.5 Influence of informational context on perceived healthiness (n = 25)

Sustainability labels and claims tend to produce more consistent effects on perceived healthiness when presented in isolation (n = 15) than when combined with additional informational cues (n = 10). With the exception of one study, all designs allowed the effect of sustainability-related cues to be isolated, either through comparisons with neutral control conditions (i.e., products without sustainability information) or across different levels of sustainability information (e.g., higher vs. lower sustainability scores), ensuring that observed effects could be attributed to the sustainability-related cue rather than to additional informational cues. The only exception was reported by Lieke et al. (2024), who conducted an online experiment with German consumers (n = 411). Participants evaluated product images that differed only in the presence of an RSPO label or a “palm-oil-free” claim, with perceived healthiness being assessed via direct comparison. Results, reported as the percentage of respondents who identified one product as healthier, showed that, for chocolate spread, the sustainability-labelled product was perceived as less healthy than the alternative with the health-related cue, indicating a negative effect.

A comparison across informational contexts suggests that the influence of sustainability-related cues becomes less predictable when consumers are simultaneously exposed to multiple sources of product information. Consistent with this pattern, studies examining sustainability labels and claims in isolation predominantly reported robust effects. These studies primarily employed randomised designs (n = 11) and were typically conducted in online settings (n = 10). Health perceptions represented the most frequently assessed outcome (n = 10), while a smaller number examined both health and nutrition perceptions (n = 4) and one examined nutrition perceptions only. In terms of effects, robust results were most frequent (n = 10; 66.67% of studies), whereas null (n = 3; 20.00%) and heterogeneous patterns (n = 2; 13.33%) were less common. One example comes from Carneiro et al. (2025), who conducted a randomised online experiment with German consumers (n = 203) manipulating the presence of a “rescued” claim on ice cream products. Participants evaluated product images, including perceived healthiness. Results showed that the sustainability claim increased perceived healthiness compared to the control condition, indicating that communicating the use of rescued ingredients can generate a positive health halo effect.

By contrast, studies examining sustainability-related cues alongside additional informational cues showed greater variability in outcomes. Randomised designs were the most common (n = 8), and all experiments were conducted in online settings. The majority of studies focused on health perceptions (n = 8), while a smaller number examined both health and nutrition perceptions (n = 2). In terms of effects, heterogeneous patterns were slightly more frequent (n = 4; 40.00% of studies), whereas robust and null results were each observed in three studies (30.00% each). This variability was evident in Jürkenbeck et al. (2024), who conducted a randomised online experiment with German consumers (n = 1061) in which product images displayed a Nutri-Score, an Eco-Score, both labels, or no label. Results showed inconsistent effects of the environmental label on health perceptions across products: perceived healthiness increased for crackers with a positive Eco-Score (A), whereas no significant effect was observed for products with a negative Eco-Score (E), such as chocolate. This suggests that the spillover of sustainability information into health perceptions depends on the valence of the environmental cue.

**Fig. 3:**
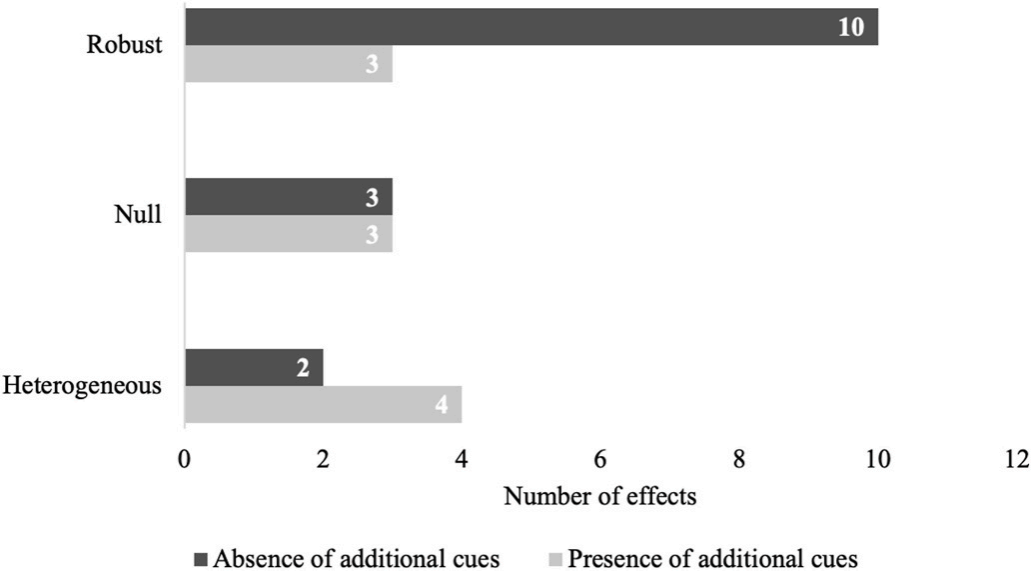
Distribution of effects by informational context of sustainability-related cues.

The distribution of effect categories across the two informational contexts is illustrated in Fig. 3.

## 4 Discussion

### 4.1 Strengths and interpretation of findings

Notwithstanding the limitations, this review has some important strengths. It provides a systematic synthesis of empirical studies examining how sustainability-related cues influence perceived food healthiness, helping to frame sustainability labels and claims as a distinct line of research rather than conflating them with nutrition labels and claims. By examining studies in which sustainability-related cues were presented both in isolation and alongside additional informational signals, the review highlights how informational context shapes observed effects and enables assessment of their independent influence on health-related perceptions.

The findings of this review are consistent with previous research showing that non-health product cues can shape consumers’ health-related judgments (Berry & Romero, 2021; Gonzales et al., 2023; Schuldt & Schwarz, 2010). In particular, sustainability-related information can lead consumers to infer greater healthiness from products associated with environmental or ethical attributes (Ruan et al., 2026; Wei et al., 2018). Importantly, this review goes beyond existing literature by showing that the effect appears more pronounced for general evaluations of product healthiness than for perceptions of specific nutritional attributes. This aligns with evidence that consumers rely on heuristic cues for overall evaluations, while more detailed nutritional inferences require additional information or cognitive processing (Eichin et al., 2025; Sproesser et al., 2023). Finally, the results highlight the role of informational context: sustainability-related cues exert a more consistent influence when presented in isolation, whereas their effects become more variable when combined with other signals, such as nutrition labels or warning messages (Marquis et al., 2023; Shaikh et al., 2024).

### 4.2 Overview of the findings

Our systematic review highlights several key findings on the relationship between sustainability-related product cues and perceived food healthiness. First, sustainability labels and claims frequently increase perceptions of healthiness, reflecting a clear health halo effect (Berry & Romero, 2021; Schuldt et al., 2012). When consumers encounter cues suggesting environmental or ethical benefits, they often generalise this positivity to the product as a whole, including its healthiness (Grummon et al., 2025; Wei et al., 2018). These inferences arise even when sustainability attributes have no nutritional relevance, indicating that consumers rely on broad, heuristic judgments rather than detailed evaluation (Paul et al., 2026). When asked whether a product seems “healthy,” consumers often draw on general impressions, making these evaluations more susceptible to heuristic spillovers from positive sustainability cues (Eichin et al., 2025). In contrast, nutrient-specific estimates (e.g., calories, fat, sodium) require more analytic processing and are therefore less easily swayed by irrelevant attributes (Büttner et al., 2024). The ambiguity of the term “healthiness” further amplifies this effect, as consumers can project their own beliefs and expectations onto the product. Motivated reasoning may also play a role: sustainability cues may align with consumers’ values or identity, increasing their willingness to see the product as healthier overall (Stanovich & West, 2000). Together, these mechanisms help explain why sustainability labels and claims more reliably affect general health perceptions than precise nutritional judgments.

Second, sustainability cues tend to exert stronger effects when presented in isolation because they face less competition from other cues. When a single sustainability message is the most salient element on the package, it draws more attention and more readily shapes overall impressions (Carneiro et al., 2025; Ruan et al., 2026). By contrast, presenting sustainability-related cues alongside nutrition labels, warnings, or other product information introduces cognitive load, requiring consumers to process multiple cues simultaneously (Jürkenbeck et al., 2024; Shaikh et al., 2024). This can dilute the influence of sustainability information or shift attention towards more familiar and established signals. Additionally, attentional salience plays a key role: isolated sustainability cues stand out more clearly, whereas in multi-label contexts they must compete with better understood nutrition information (Itti et al., 1998). Interference from established nutrition labels may reduce the likelihood that consumers rely on heuristic spillovers from sustainability cues, leading to weaker or more inconsistent halo effects (Biondi & Camanzi, 2020).

### 4.3 Research implications

This review highlights several directions for future research. First, more evidence from real-world settings is needed to assess whether the perceptual effects observed in experimental studies translate into actual consumer behaviour. As most studies rely on online experiments, research in field settings or naturalistic shopping environments could provide valuable insights into how sustainability-related cues influence real food choices. Second, future research should examine situations in which sustainability labels and claims are misaligned with a product's actual nutritional or environmental quality. Focusing on such discrepancies may help clarify how consumers interpret conflicting signals and how this affects both perceptual evaluations and behavioural responses. Finally, further research is needed to better understand the cognitive mechanisms underlying these effects, particularly how sustainability-related cues influence the weighting of product attributes in consumer decision-making. This includes examining whether health-related inferences are primarily driven by heuristic processing or more reflective evaluations of product information.

### 4.4 Policy and managerial implications

The findings of this review have important implications for food labelling policy. Sustainability-related cues can influence perceived product healthiness even when they do not reflect actual nutritional properties, raising concerns about misleading health inferences. Consumers may therefore interpret sustainability labels and claims as proxies for nutritional quality, even when no such relationship exists. This can lead to misunderstandings whereby environmental or ethical product attributes are incorrectly translated into assumptions about healthiness or nutritional quality. In this sense, sustainability-related communication can be understood as part of the commercial determinants of health, as product claims shape how food is interpreted in food environments, potentially diverging from underlying nutritional quality. When such alignment is lacking, these claims may contribute to food greenwashing by implicitly signalling healthiness where it is not warranted. The health halo effects identified in this review suggest one mechanism through which greenwashing may operate, as consumers may infer benefits that extend beyond the specific information communicated by sustainability labels and claims. The commercial incentives to use sustainability messaging are strong, and without clear regulatory standards, such claims can reinforce existing power asymmetries by allowing manufacturers to shape consumer perceptions through ambiguity rather than evidence. At the same time, these cues can serve as behavioural signals that guide consumer choices and may be leveraged in nudging policy interventions. When sustainability labels and claims align with both human and planetary health dimensions, they may support communication strategies that promote more sustainable, health-oriented consumption patterns. In this context, the regulatory asymmetry between nutrition- and sustainability-related cues is particularly evident. While nutrition claims are subject to strict, evidence-based requirements, sustainability claims remain governed by more general, less standardised approaches. Addressing this imbalance through stronger requirements for supporting evidence and clearer standardisation of sustainability labels and claims, potentially in line with emerging regulatory initiatives, could reduce misleading effects and improve transparency in food labelling. Greater regulatory harmonisation may also improve consistency across food markets and reduce consumer confusion arising from the proliferation of diverse sustainability claims and certification schemes.

### 4.5 Limitations of the review

As with all systematic reviews, our study has some important limitations. First, the heterogeneity of study designs, sustainability-related cues, and outcome measures limits the feasibility of quantitative meta-analysis and requires a narrative synthesis, as discussed above. Second, the search strategy was restricted to two major databases, Web of Science and PubMed; although these capture a large share of peer-reviewed literature, relevant studies indexed in other databases may have been missed. In addition, the search strategy incorporated nutrition- and health-labelling terminology because the literature examining the relationship between sustainability-related cues and health halo effects is situated within the fields of food labelling and nutrition. However, this approach may have favoured the identification of studies conducted within nutrition science and food-labelling research. As a result, relevant contributions from adjacent fields such as marketing, psychology, or behavioural economics, where sustainability cues may be conceptualised in terms of perception, evaluation, attitudes, heuristics, or consumer judgement, may be underrepresented. A broader set of search terms could have enabled a more comprehensive capture of the interdisciplinary evidence base. Moreover, studies focusing exclusively on organic labels and claims were excluded, as this represents a distinct research stream and was specified as an exclusion criterion before data extraction and analysis. However, organic labels often function simultaneously as signals of environmental sustainability and product healthiness, generating overlapping consumer associations across both domains (Fernqvist & Ekelund, 2014; Hughner et al., 2007; Magnusson et al., 2001). This dual role blurs the conceptual boundary between sustainability and health-related cues, making the distinction less straightforward in practice (Schuldt & Schwarz, 2010; Shaikh et al., 2024). While the exclusion was conceptually motivated, it may have led to an underestimation of the overall impact of sustainability-related cues and may have narrowed the scope of the review. This limitation is partially mitigated by the fact that the impact of organic labelling on consumer healthiness perceptions is being examined separately through a dedicated PROSPERO-registered systematic review. Furthermore, studies involving children and adolescents were excluded, limiting the generalisability of the findings to younger populations and highlighting an important area for future research. Finally, as the review focuses on perceptual outcomes related to perceived food healthiness and nutritional inferences, the extent to which sustainability labels and claims influence actual food choices or consumption remains less clearly established.

Additional limitations stem from the underlying literature. The evidence base is geographically concentrated in Europe and North America, with other regions underrepresented. Studies rely heavily on online experimental designs, with fewer laboratory experiments and very limited real-world or field evidence. Moreover, most studies examine perceptual judgments rather than behavioural outcomes, further limiting conclusions on actual food choices. Publication bias may influence the evidence base, as studies reporting significant or positive effects are more likely to be published than those finding null or inconsistent results. The predominance of positive findings should therefore be interpreted cautiously, as the true magnitude of sustainability-related health halos may be smaller than suggested. Finally, most included studies were conducted online, raising questions about ecological validity. Online experiments may either amplify these patterns by providing simplified, low-stakes decision environments or attenuate them, as participants may be less engaged than in real purchasing contexts. Regarding the methodological quality of the included studies, most adopted randomised experimental designs with appropriate statistical analyses. However, key aspects of randomisation and allocation concealment were often insufficiently reported, and outcomes were typically based on self-reported measures with limited information on reliability. Quasi-experimental studies demonstrated good comparability and analytical rigour but lacked pre–post measurements, thereby limiting causal inference. The single cross-sectional study provided more limited evidence, with some uncertainty in measurement and control of confounding.

## 5 Conclusion

This systematic review provides a structured synthesis of empirical studies examining how sustainability-related product cues influence perceived food healthiness. The available evidence suggests that sustainability labels and claims can generate a health halo effect, although the magnitude and consistency of this effect vary depending on the type of perception outcome assessed and the informational context in which these cues are presented. By clarifying how sustainability-related cues shape both general health evaluations and specific nutritional inferences, this review contributes to a more coherent understanding of how these types of cues interact with perceptions of food healthiness. As sustainability messaging becomes increasingly implemented in food environments, these findings highlight the need to consider such claims within the broader commercial determinants of health, given their potential to shape consumer judgments in ways that may not reflect actual nutritional quality. Understanding these dynamics is essential for advancing knowledge on the drivers of food choice and informing the design and regulation of food information environments at the population level. These dynamics must be addressed to ensure that market-based sustainability signals support, rather than distort, healthier and more sustainable population diets.

## Ethics approval

This study is a systematic review of previously published studies and does not involve human participants or animals. Therefore, ethical approval was not required.

## Declaration of generative AI use

During the preparation of this work, the authors used ChatGPT (OpenAI) to support language editing. After using this tool, the authors reviewed and edited the content as needed and take full responsibility for the content of the published article.

## Funding sources

This research did not receive any specific grant from funding agencies in the public, commercial, or not-for-profit sectors.

## CRediT authorship contribution statement

**Agnese Palvarini:** Conceptualization, Data curation, Formal analysis, Investigation, Methodology, Writing – original draft, Writing – review & editing. **David Stuckler:** Conceptualization, Investigation, Methodology, Supervision, Validation, Writing – review & editing. **Martin McKee:** Validation, Writing – review & editing. **Courtney McNamara:** Conceptualization, Investigation, Methodology, Supervision, Validation, Writing – review & editing.

## Declaration of competing interests

The authors of the manuscript have nothing to declare.

## Data Availability

Data sharing is not applicable to this article as no new data were created in this study.
